# Quality, reliability, and transparency of late-life depression videos on Chinese social media: a cross-sectional study of Douyin, Rednotes, and BiliBili

**DOI:** 10.3389/fpsyt.2026.1817890

**Published:** 2026-04-28

**Authors:** Xiaoyi Chen, Xuchen Luo, Jiehua Deng

**Affiliations:** 1 Department of Plastic and Aesthetic Surgery, The Second Affiliated Hospital of Guilin Medical University, Guilin, China; 2 School of Clinical Medicine, Xiangnan University, Chenzhou, China; 3 College of Business, City University of Hong Kong, Hong Kong, Hong Kong SAR, China; 4 School for Economics and Management, Zhejiang University of Science & Technology, Hangzhou, China

**Keywords:** health communication transparency, information quality, late-life depression, short-video, social media health information

## Abstract

**Background:**

Late-life depression is common in older adults and is often under-recognized. Short-video platforms have become a major source of mental health information. However, content quality and transparency remain uncertain.

**Methods:**

We conducted a cross-sectional assessment of highly viewed videos on late-life depression on three Chinese platforms. We searched each platform using the keyword in Chinese “Late-life depression”. We selected the top 200 videos by view count on Douyin, Rednotes (Xiaohongshu), and BiliBili. After exclusions, 562 videos were included (Douyin, n=188; Rednotes, n=188; BiliBili, n=186). Two medically trained raters scored videos using the Global Quality Score (GQS), modified DISCERN (mDISCERN), and JAMA benchmark criteria. We also coded content categories and creator types. We assessed platform differences using non-parametric tests. We examined associations between a limited engagement proxy, defined as the comment-to-view ratio, and quality scores using Spearman correlation.

**Results:**

Video duration differed across platforms (p<0.001). Engagement indicators were higher on Douyin and Rednotes than on BiliBili. Symptoms were the most common topic on all platforms. Prevention and intervention ranked second on Douyin and Rednotes. On BiliBili, causes and case-based analysis were also common. Overall quality was moderate. Mean GQS ranged from 2.96 to 3.05. Transparency was limited. Mean JAMA ranged from 1.91 to 2.04. Reliability was slightly higher on BiliBili based on mDISCERN. Creator type was strongly associated with scores. Expert and institutional videos scored higher than general and marketing-oriented accounts. Correlations between visible audience interaction and quality were weak.

**Conclusion:**

Highly viewed late-life depression videos on major Chinese platforms show moderate quality and limited transparency. Exposure does not reliably signal higher-quality information. Platforms and health authorities should strengthen source disclosure and promote evidence-based content from qualified creators.

## 1 Introduction

Late-life depression is a prevalent mental health condition in older adults. It is associated with functional decline, poorer quality of life, and increased healthcare use (1, 2). It also increases the risk of social isolation and suicide (3–5). These burdens extend to caregivers and health systems. The condition is often underdiagnosed (6, 7). Symptoms can be misattributed to aging or physical comorbidities (8, 9). Stigma and limited access to mental health services can further delay help-seeking.

Early recognition and evidence-based treatment can improve outcomes. Treatment options include psychotherapy, pharmacotherapy, and stepped-care models (10–12). Effective care also requires attention to comorbid chronic disease, cognitive change, and polypharmacy (13, 14). Safety is a core concern. Clinicians often stress warning signs and the need for urgent assessment when self-harm risk emerges (15). These messages are critical for the public. Yet they are not always communicated clearly outside clinical settings.

Digital media has become a major channel for health information (16, 17). Social media platforms provide rapid access to advice, personal stories, and peer support. Short-video platforms are especially influential because they compress information into easily consumed formats (18). They also combine entertainment and education. For mental health topics, this environment can be a double-edged sword. It can improve awareness and normalize help-seeking. It can also spread oversimplified or misleading claims.

Algorithmic recommendation systems shape what users see. Ranking is often driven by engagement signals such as views, likes, comments, and reposts. These signals reflect attention rather than accuracy (19, 20). Health information quality may not be rewarded. Content that is emotionally salient may spread faster. Commercially motivated creators may also exploit attention dynamics (21, 22). This is relevant for depression content. Viewers may interpret high exposure as a credibility cue. That inference can be wrong.

The quality of online health information is commonly evaluated using standardized tools. The Global Quality Score is often used to assess overall usefulness and clarity (23, 24). The modified DISCERN instrument focuses on reliability and balance of information (25, 26). The JAMA benchmark criteria assess transparency, including authorship and attribution (27, 28). These instruments have been applied in studies of medical information on YouTube and other platforms. They have also been used to evaluate content on disease prevention, treatment decisions, and public health emergencies. However, evidence remains limited for late-life depression content on major Chinese platforms. Many studies focus on other conditions, younger populations, or a single platform. Cross-platform comparisons are still scarce.

China’s social media landscape has unique features. It includes dominant platforms with distinct content formats and user communities (29–31). Douyin is optimized for short clips and rapid consumption (32). BiliBili supports longer-form video and community discussion (33). Rednotes combines lifestyle sharing with video and image-text posts (34). These differences may shape how depression information is framed and which creators gain visibility. They may also shape the balance between professional education and personal narratives. Yet empirical comparisons across these platforms remain limited.

Creator identity is another key factor. Videos posted by clinicians, researchers, hospitals, and recognized institutions may be more aligned with evidence-based knowledge (35). In contrast, general users may share subjective experiences. Marketing-oriented accounts may prioritize conversion rather than accuracy. Commercial incentives can encourage certainty and exaggeration. For mental health information, nuance matters. It is important to clarify what is known, what is uncertain, and when professional care is needed.

This study aims to strengthen evidence on late-life depression information in the Chinese social media environment. We conducted a cross-sectional assessment of videos on Douyin, Rednotes, and BiliBili retrieved using the keyword in Chinese “Late-life depression”. We evaluated each video using the Global Quality Score, modified DISCERN, and JAMA benchmark criteria. We coded content categories to describe what topics dominate public exposure. We also classified creator identity to test whether professional sources provide higher-quality information. Finally, we examined whether a visible audience interaction based on the comment-to-view ratio is associated with video scores. We expect that engagement will not reliably track quality.

Our findings have implications for public health communication and platform governance. They can inform strategies to promote source disclosure and evidence-based messaging. They can also guide clinicians and institutions in producing short, accurate, and accessible content tailored to platform norms.

## 2 Methods

### 2.1 Ethics statement

All data from Douyin, Rednotes, and BiliBili were publicly available. This study did not involve clinical records, human participants, human biological samples, or experimental animals. Therefore, in line with the principles of the Declaration of Helsinki, ethics committee approval and informed consent were not required.

### 2.2 Research design

#### 2.2.1 Video selection strategy

We selected Douyin, Rednotes, and BiliBili as representative major Chinese platforms for short-video and social-media content. We searched within each platform using the Chinese keyword “老年抑郁症”. All videos were collected on January 1, 2026, using a cross-sectional search strategy designed to capture the platform content environment at a single predefined time point. To reduce personalization, we used newly created accounts and cleared cookies and browsing data before each search (36, 37). Within each platform, we sorted results by view count in descending order. We focused on the top 200 most-viewed videos on each platform because these videos are likely to have the greatest public reach and potential influence on mental health information exposure. However, this strategy was designed to capture high-exposure content rather than the full spectrum of available videos. This yielded 600 videos in total. This approach reflects typical user exposure because it focuses on highly viewed content. It also captures videos most likely to reach audiences interested in late-life depression.

We included videos that focused on late-life depression. Topics included symptoms, identification, risk factors, and treatment or care advice. All included videos were publicly accessible. We excluded videos with only limited relevance to late-life depression, such as general mood content without an older-adult or depression focus. We also excluded content without health informational value. We removed duplicate uploads across platforms.

We classified creators into five categories. These categories were experts and health professionals, professional institutions, news media, health science communication platforms, and marketing-oriented creators. Experts and health professionals included clinicians, researchers, and healthcare staff involved in depression research or care. Professional institutions included hospitals and related healthcare organizations. News media included officially recognized media organizations in China. Health science communication platforms and marketing-oriented creators were non-professional creators. Marketing-oriented creators focused on promoting health products or paid services, or driving traffic to commercial offerings.

Video content was coded according to the main informational themes presented in each video. Specifically, videos were coded as definition-related when they primarily introduced the concept or basic characteristics of late-life depression, as symptom-related when they mainly described emotional, cognitive, behavioral, or somatic manifestations, as incidence-related when they mainly referred to prevalence or epidemiological occurrence, as cause-related when they mainly discussed determinants such as loneliness, bereavement, chronic disease, family stress, or aging-related vulnerability, as prevention-and-intervention-related when they mainly provided advice on coping, treatment, help-seeking, or symptom management, as patient self-report when the content was primarily based on personal illness narratives, and as case analysis when the video mainly used specific examples or clinical scenarios for explanation. Because a single video could contain more than one theme, multiple content codes were allowed. When a theme was only briefly mentioned but not substantively explained, it was not coded as a primary content element. This thematic classification was intended to describe the main informational focus of the videos and did not separately assess whether the content was evidence-based, balanced, or potentially harmful.

Creator type was determined according to the most authoritative identifiable account attribute. Accounts were classified as experts when the creator was a verified psychiatrist, psychologist, clinician, geriatrician, or other health professional with a clearly stated professional identity. Accounts were classified as professional institutions when they represented hospitals, clinics, universities, or official health organizations. News media referred to accounts primarily engaged in news reporting or media dissemination. Health science communication platforms referred to non-institutional accounts mainly devoted to health education or science communication. Accounts primarily oriented toward promotion of health products or paid services, or traffic generation for commercial offerings, were classified as marketing-oriented creators. When account characteristics overlapped across categories, verified professional identity or institutional affiliation was prioritized over communication style or promotional features.

#### 2.2.2 Video quality assessment

We used three standardized instruments to evaluate videos on late-life depression across Douyin, Rednotes, and BiliBili. These tools assessed overall quality, reliability, transparency. The instruments were the Global Quality Score, the modified DISCERN, the JAMA benchmark criteria (38–41).

First, the Global Quality Score measured overall quality. It used a five-point scale from 1 to 5 (**Table 1**). Higher scores indicated higher quality. The score reflected overall usefulness and clarity of presentation.

**Table 1:** GQS criteria.

Item	Description
1	Poor quality and flow, most information missing; technique misleading; unlikely to be useful for patient education
2	Generally sparse quality and flow, some information provided but many important topics missing; technique poor; of very limited use to patients
3	Moderate quality and suboptimal flow, some important information provided adequately but others poorly discussed; technique basically adequate; somewhat useful for patients
4	Good quality and generally good flow, majority of information provided but some topics not covered, technique almost adequate; useful for patients
5	Excellent quality and flow, full information provided; technique adequate; highly useful for patients

Second, the modified DISCERN measured reliability (**Table 2**). It consisted of five yes or no items. Each “yes” received 1 point. Each “no” received 0 points. Higher scores indicated higher reliability.

**Table 2:** JAMA criteria.

Criterion	Description
Authorship	Authors and contributors, their affiliations, and relevant credentials should be provided
Attribution	References and sources for all content should be listed clearly, and all relevant copyright information noted
Disclosure	Ownership, sponsorship, advertising, underwriting, commercia funding arrangements or support, or potential conflicts of interest should be prominently and fully disclosed
Currency	Dates that content was posted and updated should be indicated

Finally, the JAMA benchmark criteria assessed transparency. It covered key disclosure elements such as authorship and affiliation (**Table 3**). Each satisfied criterion received 1 point. Each unmet criterion received 0 points. The total score ranged from 0 to 4. Higher scores indicated better transparency.

**Table 3:** mDISCERN criteria.

Item	Description
1	Are the aims clear and achieved?
2	Are reliable sources of information used? (i.e., licensed psychiatrists, psychologists, geriatricians, or other relevant clinicians)
3	Is the information presented balanced and unbiased?
4	Are additional sources of information listed for patient reference?
5	Are areas of uncertainty mentioned?

To improve scoring consistency and reproducibility, we recruited two raters with medical training to independently assess all included videos. Before formal rating, both raters received standardized training on the operational definitions of each item in the Global Quality Score, modified DISCERN, and JAMA benchmark criteria, as well as the coding rules for content and creator classification. The raters then completed a pilot assessment of 20 videos to refine the coding rules and align rating standards. After independent scoring, the final score for each video was calculated as the mean of the two raters’ ratings. If disagreements arose, the two raters first re-checked the original video and discussed the disputed item. If consensus could not be reached, a third author reviewed the case and made the final decision based on the predefined coding rules.

#### 2.2.3 Statistical analysis

We analyzed the data using Stata and SPSS. Continuous variables were summarized as medians due to skewed distributions. Categorical variables were summarized as counts and percentages. Differences across the three platforms were tested using the Kruskal–Wallis test for continuous variables and the chi-square test for categorical variables. When the overall test was significant, *post hoc* pairwise comparisons were conducted using Dunn’s test with Holm adjustment. Because platform-level measures such as watch time, completion rate, and retention were not publicly available in a standardized form across the three platforms, we used the comment-to-view ratio as a limited proxy for visible audience interaction. This metric was intended to reflect relative commenting activity conditional on exposure, rather than the full multidimensional construct of user engagement or user stickiness. Associations between this metric and quality scores were examined using Spearman correlation. Inter-rater reliability for the GQS, JAMA, and mDISCERN total scores was assessed using the intraclass correlation coefficient. Because platform structures, video formats, and publicly visible engagement indicators differ across Douyin, Rednotes, and BiliBili, cross-platform comparisons of engagement-related measures were interpreted as contextual rather than strictly equivalent metric comparisons.

## 3 Results

### 3.1 Overview of the video screening process

**Figure 1:**
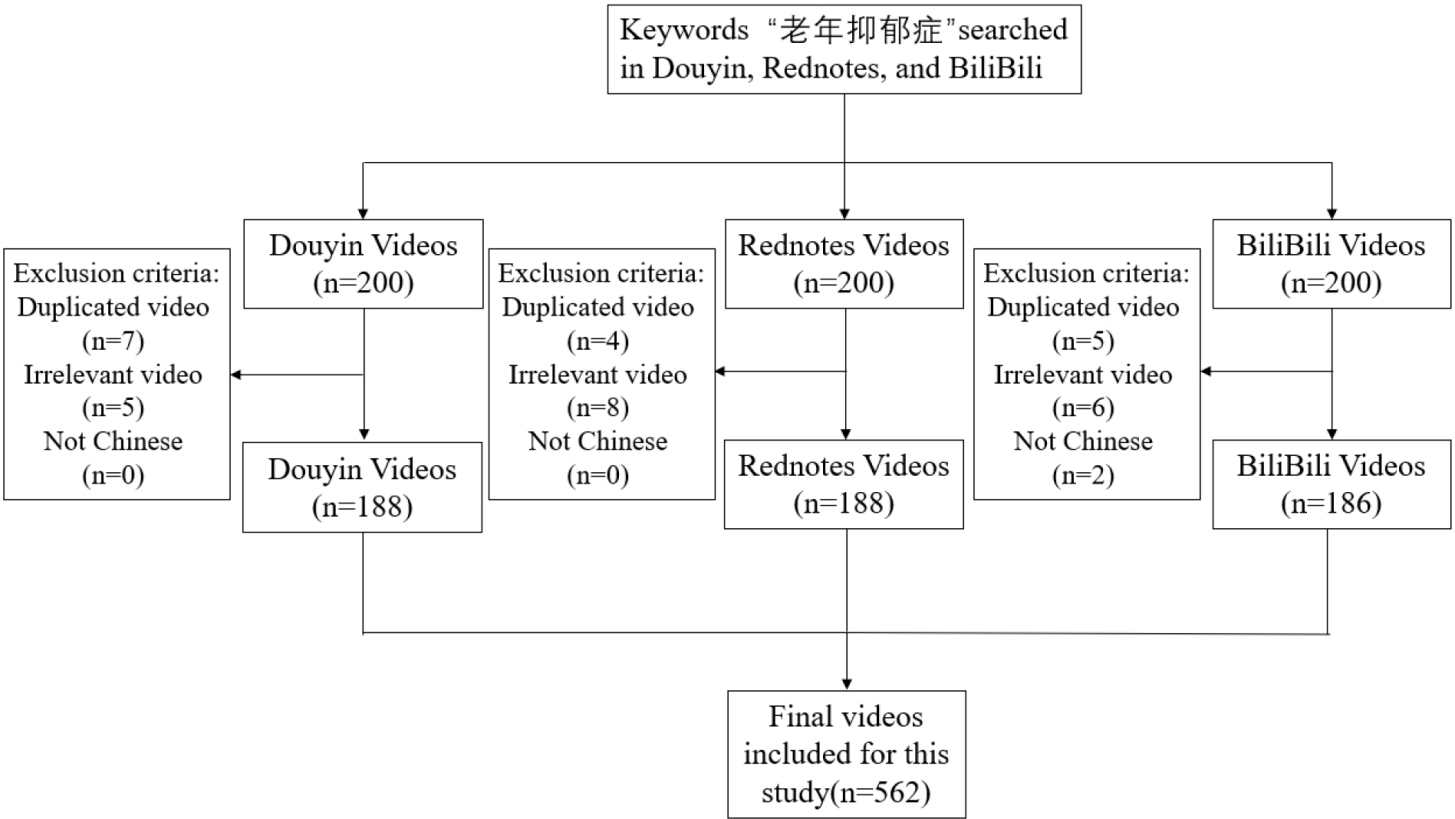
Overview of the video screening process.

We retrieved 600 videos on late-life depression from Douyin, Rednotes, and BiliBili. We excluded 38 videos in total (**Figure 1**). On Douyin, we removed 7 duplicates and 5 irrelevant videos. On Rednotes, we removed 4 duplicates and 8 irrelevant videos. On BiliBili, we removed 5 duplicates, 6 irrelevant videos, 2 non-Chinese videos, and 1 video that could not be played. The final sample included 562 videos. There were 188 videos from Douyin, 188 from Rednotes, and 186 from BiliBili.

### 3.2 Video duration and engagement

**Table 4** shows the video duration in seconds and the median values of publicly visible engagement indicators across the three platforms. We compared video duration across the three platforms. Douyin videos were much shorter than those on Rednotes and BiliBili. BiliBili videos were the longest. This pattern likely reflects platform positioning. Douyin mainly hosts short videos. BiliBili is oriented toward mid-length and long-form videos. Rednotes is a hybrid platform that supports both image-text posts and videos. Publicly visible interaction indicators were higher on Douyin and Rednotes than on BiliBili. Videos on Douyin and Rednotes showed higher counts of likes, comments, saves, and shares; however, these differences should be interpreted cautiously because engagement metrics and video formats are structurally shaped by platform-specific design and may not be strictly equivalent across platforms.

**Table 4:** Video duration and engagement characteristics across platforms.

Variable	Douyin (N = 188)	Rednotes (N = 188)	BiliBili (N = 186)	Overall p-value	Pairwise p (Holm-adjusted)
Duration seconds (Median)	132	225	489	<0.001	D vs R: <0.001; D vs B: <0.001; R vs B: <0.001
Likes (Median)	624	583	138	<0.001	D vs R: >0.05; D vs B: <0.001; R vs B: <0.001
Comments (Median)	267	241	72	<0.001	D vs R: >0.05; D vs B: <0.001; R vs B: <0.001
Shares (Median)	367	342	86	<0.001	D vs R: >0.05; D vs B: <0.001; R vs B: <0.001
Saves or Collection (Median)	186	244	48	<0.001	D vs R: >0.05; D vs B: <0.001; R vs B: <0.001

Values are presented as medians. Duration is reported in seconds. Overall p-values were obtained using the Kruskal-Wallis test. Pairwise p-values were obtained from Dunn’s *post hoc* test with Holm adjustment.

### 3.3 Content analysis of videos

**Table 5** shows the content analysis results of videos. We classified video content into seven categories. These categories covered topics such as the definition of late-life depression and its symptoms. On Douyin and Rednotes, the most common category focused on symptoms. It accounted for 35.63% and 38.30% of videos, respectively. The second most common category covered prevention and further intervention. It accounted for 31.38% on Douyin and 30.85% on Rednotes.

**Table 5:** Video content description.

Content (/)	Douyin (n, %)	Rednotes (n, %)	BiliBili (n, %)
Definition of late-life depression	55(29.26)	49(26.06)	48(25.53)
Symptoms of late-life depression	67(35.63)	72(38.30)	62(33.33)
Incidence rate	25(13.30)	28(14.89)	36(19.15)
Causes of late-life depression	42(22.34)	39(20.74)	51(27.42)
Prevention and intervention	59(31.38)	58(30.85)	49(26.06)
Patient self-reports	14(7.44)	16(8.51)	8(4.25)
Case Analysis	26(13.83)	18(9.57)	50(26.88)

Percentages do not sum to 100% because videos could be coded into multiple categories.

On BiliBili, the most common category was also symptoms, accounting for 33.33% of videos. The next most common topics differed from the other platforms. Videos on BiliBili more often discussed the causes of late-life depression and provided case-based analysis. These categories accounted for 27.42% and 26.88% of videos, respectively.

### 3.4 Creator identity

In China, health-related content on social media is subject to relatively strict regulation. Health communication that involves public safety often faces stronger oversight and may require specific qualifications.

**Figure 2:**
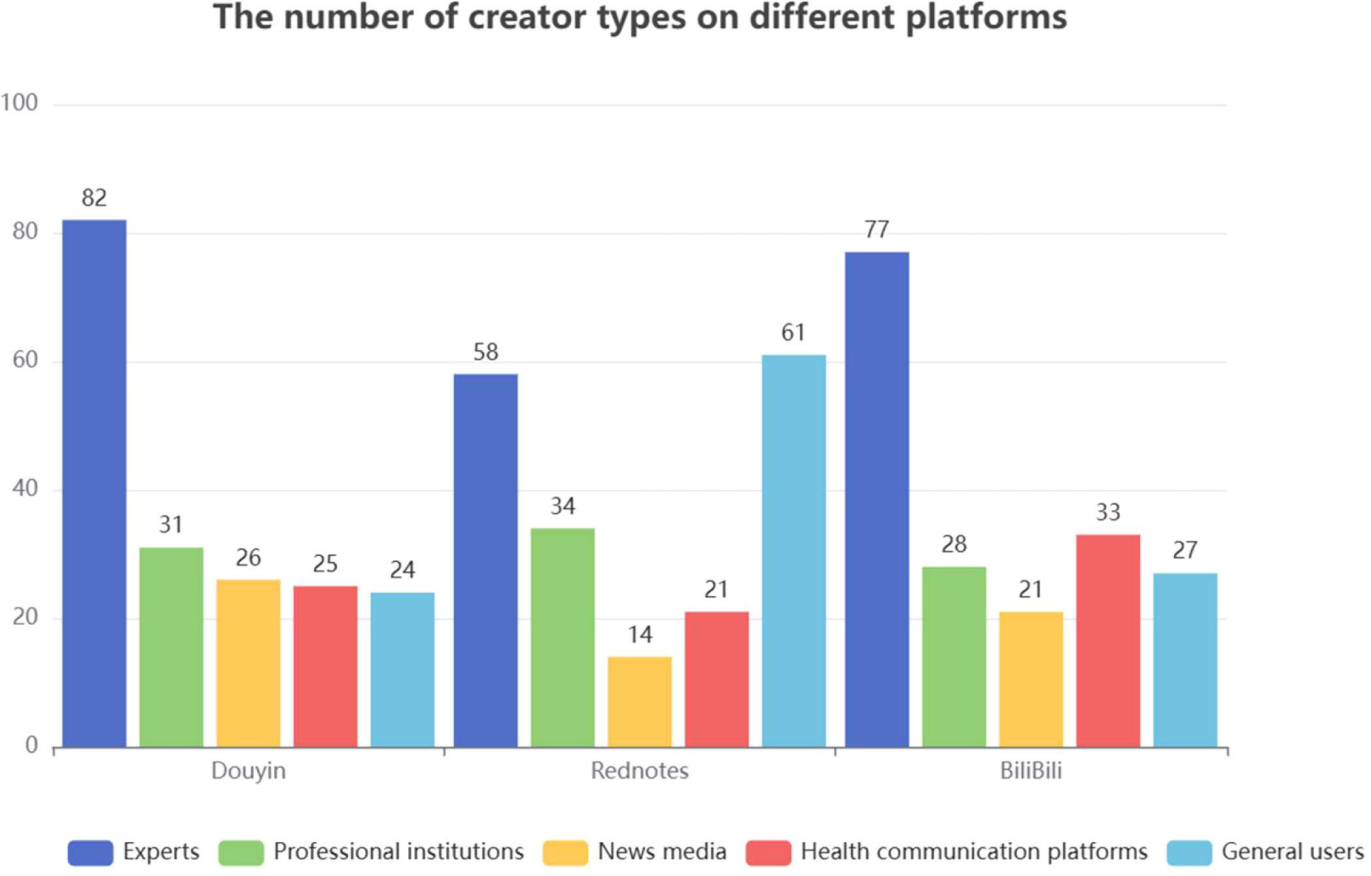
The number of creator types on different platforms.

Our results show that multiple creator groups contributed late-life depression content (**Figure 2**). However, experts posted the majority of videos across the three platforms. The remaining content came from professional institutions, news media, and health science communication platforms. Only a small share was uploaded by general users.

Overall, expert-created videos were most common on Douyin and BiliBili. On Rednotes, videos from general users were more prevalent.

### 3.5 Video quality assessment

We evaluated the included videos using the Global Quality Score, the JAMA benchmark criteria, and the modified DISCERN tool, and the results are shown in **Table 6A**. Inter-rater reliability was good to excellent across the three quality assessment instruments, with ICC values of 0.81 for GQS, 0.86 for JAMA, and 0.83 for mDISCERN. Overall, videos on all three platforms showed moderate quality based on the GQS and JAMA assessments.

**Table 6A:** Distribution of GQS, JAMA, and mDISCERN scores across platforms.

Score	Douyin (n=188)	Rednotes (n=188)	BiliBili (n=186)
Panel A-GQS			
1(very poor)	5(2.66)	5(2.66)	7(3.76)
2(poor)	47(25)	38(20.21)	42(22.59)
3(fair)	93(49.47)	96(51.06)	88(47.31)
4(good)	36(19.15)	40(21.28)	38(20.43)
5(excellent)	7(3.72)	9(4.79)	11(5.91)
Final score (Mean ± SD)	2.96 ± 0.84	3.05 ± 0.84	3.02 ± 0.91
Panel B-JAMA			
0(very poor)	7(3.72)	8(4.26)	6(3.23)
1(poor)	49(26.06)	46(24.47)	41(22.04)
2(fair)	90(47.87)	89(47.34)	91(48.92)
3(good)	38(20.21)	35(18.62)	36(19.35)
4(excellent)	4(2.13)	10(5.32)	12(6.45)
Final score (Mean ± SD)	1.91 ± 0.83	1.96 ± 0.90	2.04 ± 0.90
Panel C-mDISCERN			
0(Unreliable)	6(3.19)	8(4.26)	6(3.23)
1(Less reliable)	21(11.17)	18(9.57)	17(9.14)
2(Slightly reliable)	53(28.19)	51(27.13)	47(25.27)
3(Relatively reliable)	88(46.81)	85(45.21)	86(46.24)
4(Highly reliable)	15(7.98)	19(10.11)	22(11.83)
5(Extremely reliable)	5(2.66)	7(3.72)	8(4.30)
Final score (Mean ± SD)	2.53 ± 0.99	2.59 ± 1.06	2.67 ± 1.05

For Panels A-C, values are presented as n (%). Percentages are column percentages within each platform. Final score values are presented as mean ± SD total scores. Higher scores indicate better overall quality for GQS, better transparency for JAMA, and better reliability for mDISCERN.

For GQS, the mean score was 2.96 for Douyin, 3.05 for Rednotes, and 3.02 for BiliBili. These values suggest generally fair usefulness and clarity across platforms.

For JAMA, the mean score was 1.91 for Douyin, 1.96 for Rednotes, and 2.04 for BiliBili, indicating limited transparency in key disclosure elements.

To improve interpretability, we further examined the four individual JAMA benchmark components across platforms, as shown in **Table 6B**. Across all three platforms, authorship and currency were more frequently reported, whereas attribution and disclosure were less commonly satisfied. This indicates that the relatively low overall JAMA scores were driven mainly by limited source citation and disclosure.

**Table 6B:** Distribution of individual JAMA benchmark components across platforms.

Component	Douyin n (%)	Rednotes n (%)	BiliBili n (%)
Authorship	120 (63.83)	122 (64.89)	124 (66.67)
Attribution	62 (32.98)	65 (34.57)	69 (37.10)
Disclosure	54 (28.72)	56 (29.79)	61 (32.80)
Currency	123 (65.43)	126 (67.02)	125 (67.20)

Values are presented as n (%). Percentages are column percentages within each platform. Each JAMA benchmark component was coded as present or absent.

The mDISCERN results showed clearer platform differences. BiliBili achieved higher reliability scores than Rednotes and Douyin. Douyin had the lowest reliability.

### 3.6 Association between creator type and video scores

Creator type was closely related to the quality measures. **Table 7** shows the scores for different types of creators. Videos posted by experts and health professionals, as well as professional institutions, received higher GQS, mDISCERN, and JAMA scores than those posted by general users and marketing-oriented accounts. Marketing-oriented videos showed the lowest reliability and transparency scores. They also appeared more likely to contain promotional or exaggerated content.

**Table 7:** Mean scores of GQS, JAMA, and mDISCERN by creator type.

Categories	Experts	Professional institutions	News media	Health communication platforms	General users
GQS (Mean ± SD)	3.52 ± 0.74	3.48 ± 0.71	3.12 ± 0.69	2.24 ± 0.63	1.96 ± 0.58
JAMA (Mean ± SD)	3.07 ± 0.76	2.97 ± 0.73	2.64 ± 0.70	1.65 ± 0.61	1.03 ± 0.52
mDISCERN (Mean ± SD)	3.24 ± 0.83	3.12 ± 0.79	2.75 ± 0.75	2.19 ± 0.68	1.83 ± 0.61

Values are presented as mean ± SD total scores for each creator category. Higher scores indicate better overall quality for GQS, better transparency for JAMA, and better reliability for mDISCERN.

### 3.7 Correlations between engagement and quality

We used Spearman’s rank correlation to examine the association between the comment-to-view ratio, treated here as a limited proxy for visible audience interaction, and video quality scores. The results are shown in **Table 8**. The correlations were small in magnitude, and no statistically significant association was observed between visible audience interaction and the three score measures. These findings suggest that greater observable commenting activity relative to exposure does not necessarily indicate higher-quality information.

**Table 8:** Spearman correlation between visible audience interaction and video quality scores.

Item	GQS	JAMA	mDISCERN
Visible audience interaction	0.318(0.067)	0.251(0.084)	0.298(0.182)

Values are Spearman’s rho, with two-sided p-values shown in parentheses.

## 4 Discussion

This study assessed highly viewed videos on late-life depression across Douyin, Rednotes, and BiliBili. We focused on content that users were most likely to encounter. Overall, the information quality was moderate. Transparency was limited. Reliability varied across platforms and creator types.

### 4.1 Principal findings

Several findings are consistent across platforms. Symptoms were the most frequent topic. Prevention and intervention were also common on Douyin and Rednotes. BiliBili showed more content on causes and case-based analysis. This suggests that platforms differ in how they package mental health information. It also suggests that users may receive different messages depending on where they search.

Quality scores clustered around the mid-range. The mean GQS values were close to 3 on all platforms. This indicates “fair” usefulness at best. JAMA scores were low. This implies weak disclosure of authorship, affiliation, and sourcing. mDISCERN scores showed clearer differences. BiliBili performed slightly better. Douyin was the lowest.

### 4.2 Platform context and cross-platform differences

Platform context likely shapes format, exposure patterns, and observable interaction, and should therefore be considered when interpreting cross-platform differences in content quality and audience response. Douyin is optimized for short viewing sessions. Creators may compress complex clinical information into brief advice. This increases the risk of missing key qualifiers. It may also reduce opportunities for source attribution. BiliBili supports longer videos. Longer formats can provide more context and explanation. They may also allow step-by-step guidance. Still, length is not equal to quality. A long video can also repeat myths or present unbalanced claims.

Rednotes is a hybrid platform. It supports image-text posts and videos. It often hosts lifestyle-oriented narratives. This may attract more user-generated health stories. It can improve relatability. It can also increase variability in accuracy.

### 4.3 The role of creator identity

Creator identity was a strong determinant of information quality. Expert and institutional videos scored higher across measures. General-user and marketing-oriented content scored lower. Marketing-oriented videos also contained promotional or exaggerated claims more often. This pattern is expected. Commercial incentives can reward certainty and simplicity. Mental health topics often require nuance. They require uncertainty disclosure. They require clear boundaries on self-management advice.

These findings support stronger credential signaling. Platforms can display verification status more prominently. Platforms can also require disclosure statements for health content. This includes qualifications and institutional affiliation. It also includes whether the content is sponsored.

### 4.4 Comment-based interaction is a weak proxy for quality

Observable comment-based interaction was higher on Douyin and Rednotes than on BiliBili for this topic. Yet the correlation between the comment-to-view ratio and quality scores was weak. Because this metric captures only one visible dimension of interaction, the present findings should not be interpreted as evidence that all forms of user engagement are unrelated to content quality. This is important. It suggests that attention does not track accuracy or transparency. Recommendation systems may amplify content that is easy to consume. They may also amplify emotionally salient narratives. For late-life depression, this mismatch can matter. It may delay professional evaluation. It may normalize persistent symptoms. It may increase reliance on non-evidence-based remedies.

### 4.5 Practical implications

Platforms can reduce risk using simple governance tools. They can require basic source citation when clinical claims are made. They can add labels that distinguish professionals from non-professionals. They can flag commercial intent and sponsorship. They can also add prompts that encourage help-seeking when depression-related keywords appear. For example, prompts can recommend psychiatric evaluation when symptoms persist. Prompts can also remind viewers to seek urgent help for suicidal ideation.

Clinicians and public health agencies can also act. They can produce short, standardized videos with key messages. They can focus on symptom recognition, treatment options, and safe help-seeking. They can link to trusted resources. They can adapt content to platform norms without losing core accuracy.

### 4.6 Limitations and future research

This study has several limitations. First, we sampled only the most-viewed videos at a single time point using a cross-sectional search strategy. Because platform recommendation algorithms, user engagement patterns, and content popularity are dynamic, the set of highly viewed videos may vary over time. Therefore, our findings should be interpreted as a snapshot of the late-life depression information environment on the three platforms at the time of data collection rather than as a temporally stable representation of all available content. Second, by focusing on the most-viewed videos, the study may underrepresent lower-exposure content, including educational videos that may be of higher quality but receive less algorithmic visibility or user engagement, thereby introducing selection bias toward popularity-driven content. Third, our operationalization of audience interaction was limited. We used the comment-to-view ratio as a visible proxy for engagement, but this single indicator cannot capture the full multidimensional nature of user interaction. In addition, engagement metrics differ across platforms and are shaped by platform-specific design, recommendation logic, and content format, so cross-platform differences in visible interaction should be interpreted cautiously. We also did not measure watch time, completion rate, retention, or the sentiment and thematic content of comments, all of which may influence the reach, interpretation, and impact of health information. Fourth, we did not directly verify the factual or clinical accuracy of individual statements against established clinical guidelines. Although GQS, mDISCERN, and JAMA assess quality, reliability, and transparency, they do not provide a direct measure of medical correctness. In addition, our content classification was thematic and did not distinguish between evidence-based and non-evidence-based information, balanced and potentially misleading content, or safe and potentially harmful recommendations. Finally, we did not assess viewer outcomes, so we could not determine whether exposure to higher- or lower-quality videos influenced knowledge, attitudes, or help-seeking behaviors. Future studies could strengthen temporal robustness and external validity by conducting repeated searches across multiple time points or adopting a longitudinal sampling design, improve representativeness through stratified or random sampling across exposure strata, incorporate multiple engagement indicators and comment-level sentiment or thematic analysis, and evaluate factual accuracy using expert checklists or established clinical guidelines.

## 5 Conclusion

Highly viewed videos on late-life depression across major Chinese platforms showed moderate quality and limited transparency. Platform differences were evident. Creator identity mattered. Expert and institutional videos performed better. Marketing-oriented content performed worse. User engagement did not reliably indicate higher-quality information. Platforms should strengthen disclosure and governance for health content. Health authorities should expand evidence-based communication tailored to short-video environments.

## Data Availability

The original contributions presented in the study are included in the article/supplementary material. Further inquiries can be directed to the corresponding authors.
